# Leukemic presentation of extranodal NK/T‐cell lymphoma

**DOI:** 10.1002/jha2.245

**Published:** 2021-05-31

**Authors:** Safina Hafeez, Joseph A. DiGiuseppe

**Affiliations:** ^1^ Department of Pathology & Laboratory Medicine Hartford Hospital Hartford Connecticut USA

A 44‐year‐old woman with a 2‐ to 3‐day history of weakness, shortness of breath, and fever to 40.6^o^ C was transferred to our facility with hypoxemia refractory to mechanical ventilation, and near‐diffuse bilateral airspace opacity. Within a week of presentation, she had developed hemophagocytic lymphohistiocytosis, and her WBC count had risen from 9,900 to 73,400/μl. Review of the peripheral smear revealed a population of abnormal lymphoid cells with coarse azurophilic cytoplasmic granules, which varied in size, nuclear shape, chromatinic dispersion, and nucleolar prominence (Figure 1, 100 objective). By flow cytometry, these cells were CD8+ T cells with abnormally diminished or absent surface‐membrane CD3, and NK‐cell‐associated antigen expression (CD2+/cyCD3ε+/CD4‐/CD5‐/CD7+/CD8+/CD11b+/CD16‐/CD30‐/CD34‐/CD38+/CD56+/TdT‐). Clonal T‐cell receptor gene rearrangement and a 42.5 MB deletion in 6q (6q16.3q24.2) were demonstrated by PCR and cytogenomic microarray, respectively. The overall findings support leukemic presentation of extranodal NK/T‐cell lymphoma, in which 6q deletions are recurrent, and which may progress to an aggressive leukemia. The presence of azurophilic cytoplasmic granules is a morphologic clue to the cytotoxic nature of the abnormal lymphoid cells and should raise the consideration of a cytotoxic T‐cell or NK‐cell lymphoproliferative disorder. The patient was stabilized with dexamethasone, but at last follow‐up remained too debilitated to begin definitive chemotherapy.

## CONFLICT OF INTEREST

The authors declare no conflict of interest.

**FIGURE 1:**
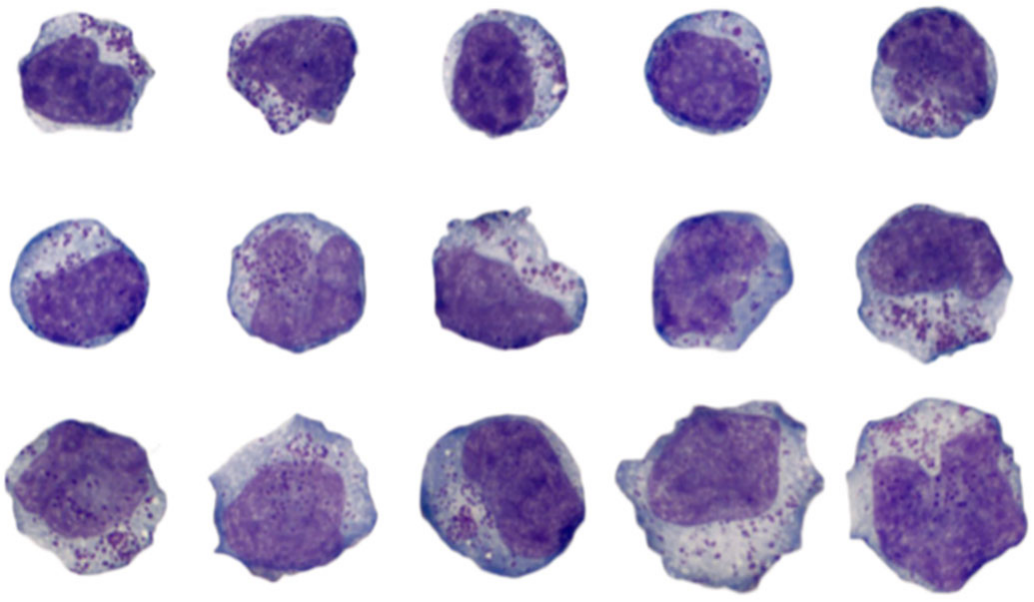
Abnormal lymphoid cells with coarse azurophilic cytoplasmic granules

